# Simultaneous saccharification and fermentation of steam-exploded corn stover at high glucan loading and high temperature

**DOI:** 10.1186/s13068-014-0167-x

**Published:** 2014-12-04

**Authors:** Zhi-Hua Liu, Lei Qin, Jia-Qing Zhu, Bing-Zhi Li, Ying-Jin Yuan

**Affiliations:** Key Laboratory of Systems Bioengineering (Ministry of Education), Tianjin University, 92 Weijin Road, Nankai District Tianjin, 300072 China; SynBio Research Platform, Collaborative Innovation Center of Chemical Science and Engineering (Tianjin), School of Chemical Engineering and Technology, Tianjin University, 92 Weijin Road, Nankai District Tianjin, 300072 China

**Keywords:** Corn stover biomass, High glucan loading, High temperature, Simultaneous saccharification and fermentation (SSF), Surfactant, Mass balance

## Abstract

**Background:**

Simultaneous saccharification and fermentation (SSF) is a promising process for bioconversion of lignocellulosic biomass. High glucan loading for hydrolysis and fermentation is an efficient approach to reduce the capital costs for bio-based products production. The SSF of steam-exploded corn stover (SECS) for ethanol production at high glucan loading and high temperature was investigated in this study.

**Results:**

Glucan conversion of corn stover biomass pretreated by steam explosion was maintained at approximately 71 to 79% at an enzyme loading of 30 filter paper units (FPU)/g glucan, and 74 to 82% at an enzyme loading of 60 FPU/g glucan, with glucan loading varying from 3 to 12%. Glucan conversion decreased obviously with glucan loading beyond 15%. The results indicated that the mixture was most efficient in enzymatic hydrolysis of SECS at 3 to 12% glucan loading. The optimal SSF conditions of SECS using a novel *Saccharomyces cerevisiae* were inoculation optical density (OD)_600_ = 4.0, initial pH 4.8, 50% nutrients added, 36 hours pre-hydrolysis time, 39°C, and 12% glucan loading (20% solid loading). With the addition of 2% Tween 20, glucan conversion, ethanol yield, final ethanol concentration reached 78.6%, 77.2%, and 59.8 g/L, respectively, under the optimal conditions. The results suggested that the solid and degradation products’ inhibitory effect on the hydrolysis and fermentation of SECS were also not obvious at high glucan loading. Additionally, glucan conversion and final ethanol concentration in SSF of SECS increased by 13.6% and 18.7%, respectively, compared with separate hydrolysis and fermentation (SHF).

**Conclusions:**

Our research suggested that high glucan loading (6 to 12% glucan loading) and high temperature (39°C) significantly improved the SSF performance of SECS using a thermal- and ethanol-tolerant strain of *S. cerevisiae* due to the removal of degradation products, sugar feedback, and solid’s inhibitory effects. Furthermore, the surfactant addition obviously increased ethanol yield in SSF process of SECS.

## Background

Environmental and economical sustainability benefits have increased the interests in alternative sources of energy [1,2]. Lignocellulosic ethanol (LCE) is considered as an important renewable alternative to fossil fuels due to the reduction of greenhouse gas emissions [3-5]. Corn stover (CS), which is the most abundant renewable resource, has been identified as one of the most promising feedstocks to produce LCE [6,7]. Generally, LCE production requires the following three major processes: pretreatment, enzymatic hydrolysis, and fermentation. Pretreatment is a necessary step for breaking down the lignin-carbohydrate complex (LCC) structures, which increases cellulose accessibility to enzymes in hydrolysis and improves ethanol yield in fermentation [8-10]. Due to the potentials for lowering environmental impact and lessening hazardous chemicals use, steam explosion pretreatment (SEP) is one of the most widely employed and efficient pretreatments for biomass refining [4,11]. SEP partially depolymerizes hemicellulose, relocates part of the lignin onto the surface of the biomass solid, and creates a large enzyme-accessible surface area [12,13]. Steam-exploded biomass was found to be highly digestible and highly fermentable, and it should be suitable for bio-based products refining [4,14,15].

Compared with separate hydrolysis and fermentation (SHF), simultaneous saccharification and fermentation (SSF) is usually preferred in LCE industry processes due to the low cost, the reduced contamination risk, and lower sugar inhibitory effects [16,17]. However, there are still several concerns about the SSF process, such as the optimum temperature discrepancies between saccharolytic enzymes and fermentation microbes [17]. In addition, one of the bottlenecks for commercialization of LCE refers to the low sugar concentration after enzymatic hydrolysis with associated low ethanol concentration in the fermentation broth [18-20]. From an economic feasibility standpoint, a high solid loading with satisfactory sugar and ethanol yields is required to reduce the cost of ethanol distillation in the downstream process of biomass refining. However, with the increase of solid loading in SSF, the concentration of inhibitors also increase, such as acetic acid, furfural, 5-hydroxymethyl furfural (HMF), and phenolic lignin degradation products (DPs) formed in pretreatment. High inhibitor concentrations may severely hamper the performance of the fermenting microorganism and, in the worst case scenario, result in a non-fermentable hydrolyzate [21-23]. Mass transfer is another inherent issue in SSF with high solid loading [14,19]. Therefore, in-depth investigation of the SSF process at high solid loading is helpful for commercialization of LCE.

In this study, two different biomass conversion processes, SHF and SSF, were investigated. Fermentation conditions that might affect the SSF performance (including inoculation optical density (OD), nutrients, initial pH, and pre-hydrolysis time) were optimized. Both washed steam-exploded corn stover (SECS) and whole slurry (with all the inhibitors present) were used to evaluate the DPs’ inhibitory effect in hydrolysis and fermentation process. Meanwhile, the SSF of SECS at high glucan loading was carried out using a novel yeast strain, thermal- and ethanol-tolerant *Saccharomyces cerevisiae*, and the surfactants (Tween 20, Tween 80, and bovine serum albumin (BSA)) were added to improve the fermentation performance. Mass balance around the whole process, including pretreatment, enzymatic hydrolysis, and fermentation, was performed to evaluate the CS biomass conversion performance.

## Results and discussion

### Enzymatic hydrolysis

SEP was adopted to break down the structure of the lignocellulosic matrix to facilitate hydrolysis and fermentation. Table 1 shows that SEP dissolved the most xylan and removed all the acetyl from untreated corn stover (UCS), which meant that the LCC structure of CS was disrupted. Although the lignin content increased, the glucan content of SECS increased by 86.1%, compared with that of UCS. In general, the high glucan content was beneficial to the bioconversion process.

**Table 1:** **Compositions of untreated corn stover and steam-exploded corn stover and degradation products in steam-exploded liquor**

**UCS (% of dry weight)**		**SECS (% of dry weight)**		**DPs in SEL (g/L)**	
Glucan	31.7 (1.1)	Glucan	59.0 (1.2)	Formic acid	2.3 (0.2)
Xylan	17.1 (0.7)	Xylan	8.5 (0.9)	Acetic acid	2.7 (0.3)
Acetyl	2.90 (0.1)	Acetyl	0	HMF	0.7 (0.1)
Lignin	12.6 (0.6)	Lignin	23.1 (1.5)	Furfural	1.0 (0.2)

DPs, Degradation products; HMF, 5-hydroxymethyl furfural; SECS, Steam-exploded corn stover; SEL, Steam-exploded liquor; UCS, Untreated corn stover. Standard deviations are shown in parentheses.

**Figure 1:**
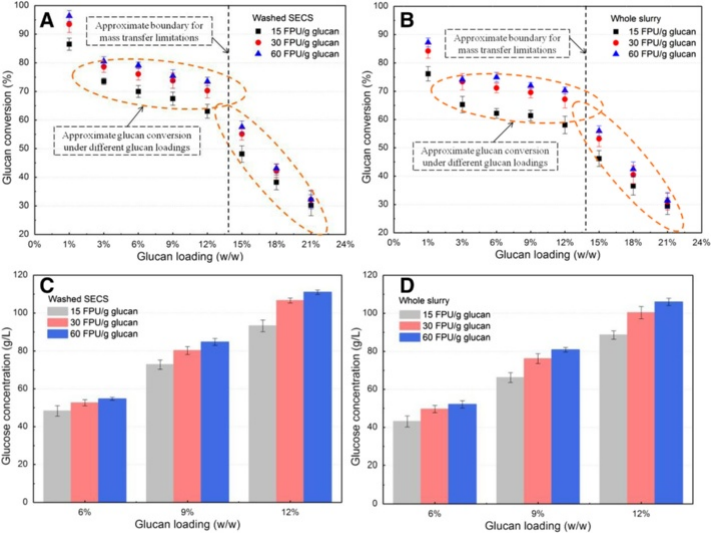
**Enzymatic hydrolysis of washed SECS and whole slurry under different glucan loadings and enzyme loadings.** Enzymatic hydrolysis conditions: 50°C, 200 rpm, and 168 hours. Glucan conversion of wash SECS **(A)** and whole slurry **(B)** and glucose concentration in enzymatic hydrolysis of wash SECS **(C)** and whole slurry **(D)** were determined. FPU, filter paper unit; SECS, steam-exploded corn stover.

CS biomass pretreated by SE was subsequently hydrolyzed at 1 to 21% (w/w) glucan loading, corresponding to 1.7 to 35.6% (w/w) solid loading (Figure 1). The results show that higher enzyme loading resulted in higher glucan conversion of either washed SECS (Figure 1A) or whole slurry (Figure 1B). The glucan conversion at 15 filter paper units (FPU)/g glucan was about 7 to 10% for washed SECS and 9 to 13% for whole slurry, respectively, lower than that at 60 FPU/g glucan, with the glucan loading varying from 1 to 15%. However, the glucan conversion at 30 FPU/g glucan was only 2 to 4% for washed SECS and 1 to 5% for whole slurry, respectively, lower than that at 60 FPU/g glucan, with the glucan loading varying from 1 to 15%. It should be noticed that glucan conversion was approximate at 18% and 21% glucan loading for all enzyme loadings, which indicated that the enzyme loading is not the main barrier for improving the enzymatic hydrolysis performance beyond 18% glucan loading. From an economic point of view, higher enzyme use should lead to higher cost of process. Actually, the enzyme accounts for a large proportion of the capital cost of industrial LCE production. Therefore, enzymatic hydrolysis of CS biomass should be the balance of the cost of enzyme and the hydrolysis performance.

The results also show that glucan conversion decreased with increasing glucan loading at all enzyme loadings (Figure 1A and 1B). But, it was interesting to note that the approximate glucan conversion was obtained for either washed SECS or whole slurry with glucan loading varying from 3 to 12% at all enzyme loadings. The previous study also reported that glucan conversion hardly changed with initial insoluble pretreated CS solids, varying from 2.5 to 25% after 168 hours hydrolysis [24]. This phenomenon indicated that the poor mixture efficiency was not obvious in enzymatic hydrolysis of SECS at 3 to 12% glucan loading. Glucan conversion of washed SECS was maintained at approximately 63 to 70% at 15 FPU/g glucan, 71 to 79% at 30 FPU/g glucan, and 74 to 82% at 60 FPU/g glucan, with glucan loading varying from 3 to 12%. However, glucan conversion of SECS was not more than 83% at 3 to 12% glucan loading, with an enzyme loading of 60 FPU/g glucan. This upper limit was likely due to the steric obstruction of glucan by other components in SECS, such as recondensed lignin and retained xylan [24,25]. Another interesting result from this data was that glucan conversion obviously decreased from about 60 to 30% for both washed SECS and whole slurry with glucan loading increasing from 15 to 21%. The one reason for this result may be that sugar concentration was rapidly increased when glucan loading was more than 15%, and hence the products’ feedback (sugar) inhibitory effect on enzymes should be strengthened [18,19]. Another reason is that the slurry’s rheological property was changed, and convective diffusion was turned into molecular diffusion, hence the boundary for mass transfer limitations may have been reached, due to the solid’s effects [19,20].

Figure 1A and 1B also shows that the DPs’ inhibitory effect on enzymatic hydrolysis decreased with the increase of glucan loading. Glucan conversion of whole slurry decreased by 6 to 9% at 15 FPU/g glucan and 3 to 5% at 30 FPU/g glucan and 60 FPU/g glucan, respectively, with glucan loading increasing from 3 to 12%, compared with that of washed SECS. This results suggested that the DPs’ inhibitory effect was obvious for low enzyme loading. However, glucan conversion of whole slurry decreased by only 1 to 2% at all enzyme loadings, with glucan loading increasing from 15 to 21%, compared with that of washed SECS. The reason for this result may be that the mass transfer limitations should be the major problem when the glucan loading is beyond 15%. These results suggested that the DPs’ inhibitory effect was mild in enzymatic hydrolysis of SECS with an enzyme loading of 30 FPU/g and 60 FPU/g glucan, especially at high glucan loading.

The ethanol concentration in the broth entering the distillation should be above 40 g/L in order to make an economical feasible process for industrial bioethanol production [18,25,26], which means that glucose concentration should be more than 80 g/L. Figure 1C and 1D show that glucose concentration reached 106.6 g/L for washed SECS and 100.3 g/L for whole slurry at 30 FPU/g glucan, and 111.2 g/L for washed SECS and 106.1 g/L for whole slurry at 60 FPU/g glucan, for 12% glucan loading hydrolysis. Enzymatic hydrolysis at high glucan loadings also saves production time and equipments and improved the production productivity, and hence decreases the capital cost of process compared with that at low- or moderate-glucan loadings. Therefore, SECS can be considered as one of the best biomasses available for bioethanol production at high glucan loading due to the small difference in glucan conversion among 6% and 12% glucan loading, the satisfied sugar concentration, and the low DPs’ inhibitory effect.

### Optimization of simultaneous saccharification and fermentation conditions

**Figure 2:**
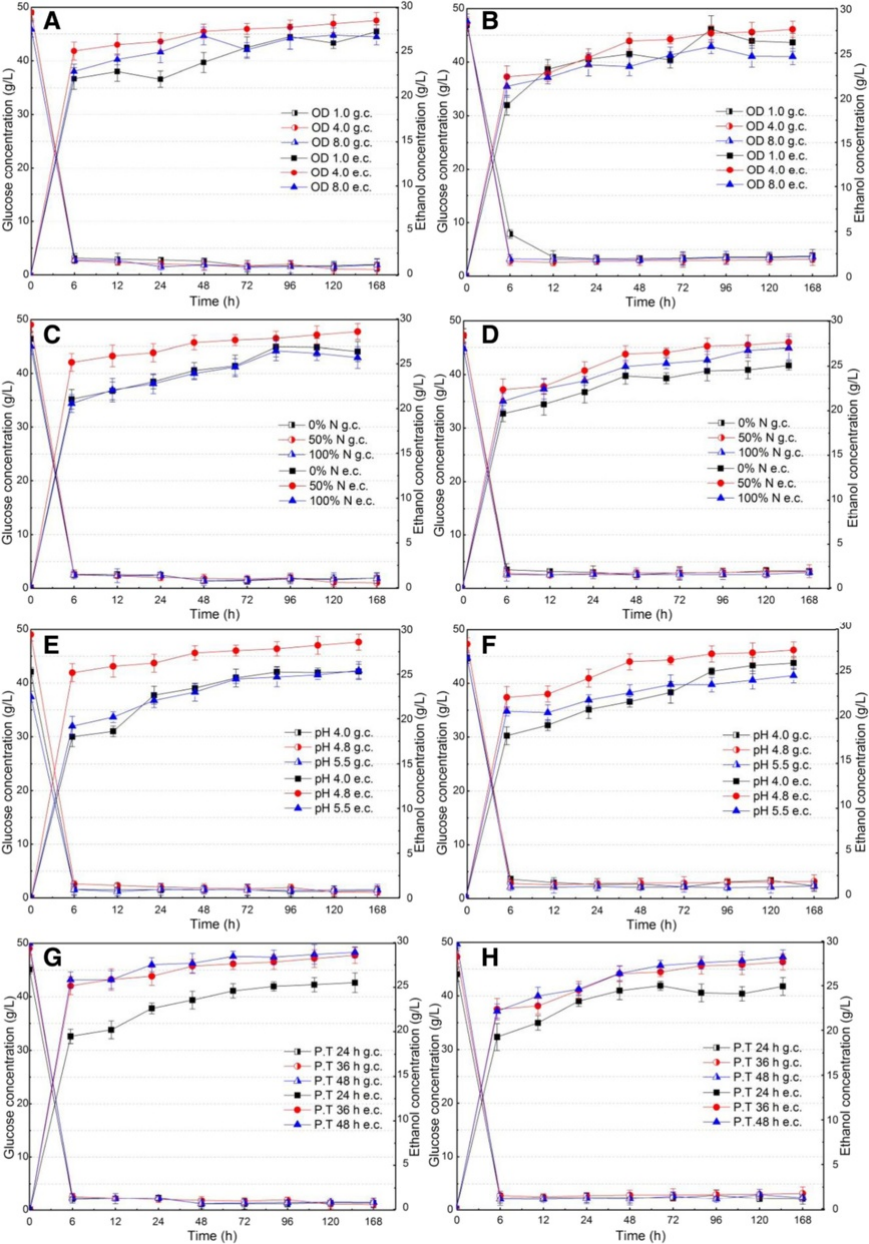
**Effects of inoculation OD (A and B), nutrients (C and D), initial pH (E and F), and pre-hydrolysis time (G and H) on the SSF performance of washed steam-exploded corn stover (A, C, E, and G) and whole slurry (B, D, F, and H).** e.c., ethanol concentration; g.c., glucose concentration; CFU, colony forming unit; N, nutrients; OD, optical density; P.T., pre-hydrolysis time; SECS, steam-exploded corn stover; WS, whole slurry.

**Figure 3:**
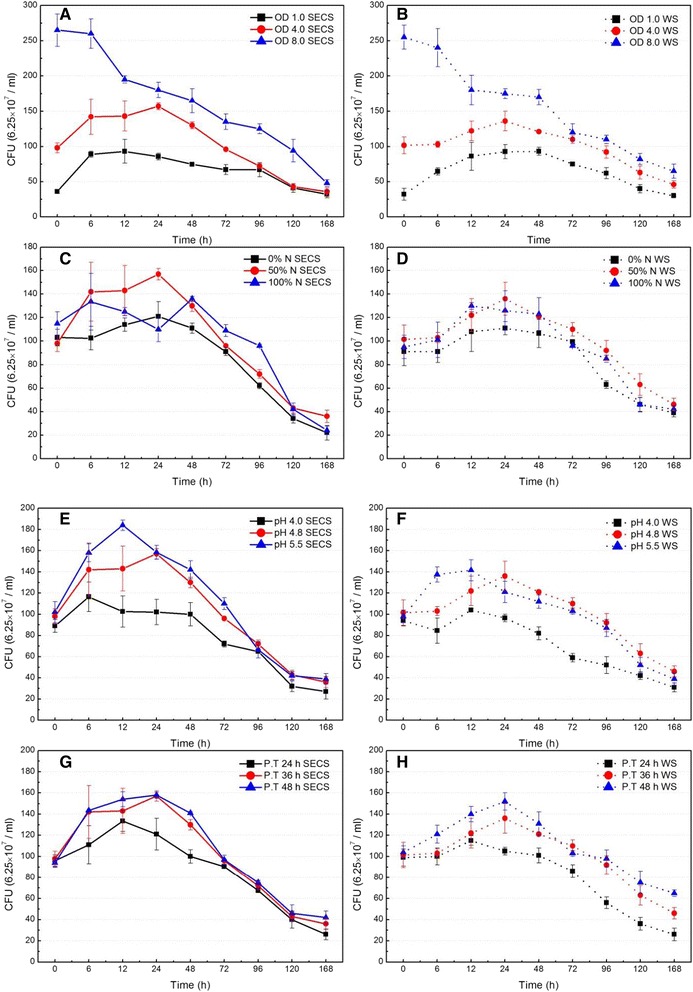
**Effects of inoculation OD (A and B), nutrients (C and D), initial pH (E and F), and pre-hydrolysis time (G and H) on cell viability (colony forming unit) in simultaneous saccharification and fermentation of washed steam-exploded corn stover (A, C, E, and G) and whole slurry (B, D, F, and H).** CFU, colony forming unit; N, nutrients; OD, optical density; P.T., pre-hydrolysis time; SECS, steam-exploded corn stover; WS, Whole slurry.

The SSF of washed SECS and whole slurry under different conditions, including inoculation OD, nutrients, initial pH, and pre-hydrolysis time, were performed at 6% glucan loading (Table 2), and the results are given in Figures 2 and 3.

**Table 2:** **Experiment design for the effects of inoculation OD, nutrients, initial pH, pre-hydrolysis time, and temperature on the SSF performance of washed SECS and whole slurry by** ***Saccharomyces cerevisiae***

**Parameter**	**Effect of OD**	**Effect of nutrients**	**Effect of initial pH**	**Effect of pre-hydrolysis time**	**Effect of temperature**
Initial OD_600_	1.0, 4.0, 8.0	4.0	4.0	4.0	4.0
Nutrients	50%	0%, 50%, 100%	50%	50%	50%
Initial pH	4.8	4.8	4.0, 4.8, 5.5	4.8	4.8
Pre-hydrolysis time (hours)	36	36	36	24, 36, 48	36
Temperature (°C)	36	36	36	36	30, 33, 36, 39, 42

100% nutrients was defined as 10 g/L yeast extract and 20 g/L peptone; 50% nutrients was defined as 5 g/L yeast extract and 10 g/L peptone; 0% nutrients was defined as 0 g/L yeast extract and 0 g/L peptone. OD, optical density; SSF, simultaneous saccharification and fermentation; SECS, steam-exploded corn stover.

The larger inoculation OD should certainly have helped to initiate the SSF process, but it resulted in the higher capital cost of seed preparation. Therefore, the optimal experiments were carried out at 36°C with 50% nutrients, initial pH 4.8, and 36 hours pre-hydrolysis to balance the cost of seed preparation and the influences of inoculation OD on the SSF performance. The results show that ethanol concentration rapidly increased at the first 6 hours under different inoculation OD, and the initial ethanol yield increased according to the order of OD_600_ = 4.0, followed by OD_600_ = 8.0, followed by OD_600_ = 1.0, for both washed SECS and whole slurry (Figure 2A and 2B). Final ethanol concentration (168 hours) displayed a similar trend in SSF, which indicated that the excessive low or high inoculation OD was adverse for SSF. The possible reason for the poor SSF performance with low inoculation OD could be that the yeast cell viability was inhibited by the solid’s inhibitory effect due to the binding of the yeast cells to the fermentation solid residues [27,28]. Figure 3A and 3B shows that the cell viability (colony forming unit (CFU)) at OD_600_ = 1.0 was lowest, which slightly increased before 12 hours and then decreased, with fermentation time varying from 24 to 168 hours, which agrees with the above analysis. As for high inoculation OD, the excessive high OD should consume more glucose and convert nutrition into the microbial biomass, resulting in low ethanol productivity. Figure 3A and 3B shows that the cell viability (CFU) at OD_600_ = 8.0 was highest, which supports the above analysis. In addition, the cell viability (CFU) at OD_600_ = 8.0 decreased by 82% for washed SECS and 75% for whole slurry at 168 hours. These results suggest that, besides the solid’s inhibitory effect, the exhaustion of nutrition should also restrict the growth of yeast at the late stage of fermentation. Therefore, combined with the analysis of initial ethanol yield, final ethanol concentration, and cell viability, the optimal inoculation OD was determined as 4.0 in SSF.

The ethanol production efficiency by *S. cerevisiae* was strongly affected by the nitrogen source in the medium in SSF [29,30]. Effect of nutrients on the SSF performance was carried out (Figure 2C and 2D). It is interesting to note that nutrients were not added under 0% nutrients (N) in SSF, but cell viability (CFU) slowly increased before 24-hour fermentation (Figure 3C and 3D), and glucose was still converted into ethanol with fermentation progression. This may be due to the fact that CS contained nutrients (such as protein and inorganic nitrogen), which were beneficial to yeast growth. It was clear that an initial ethanol yield under 50% N increased by 19.8% for washed SECS, and 13.7% for whole slurry, compared with that under 0% N (Figure 2C and 2D). Final ethanol concentration (168 hours) under 50% N increased by 8.7% for SECS, and 10.6% for whole slurry, compared with that under 0% N. Meanwhile, cell viability (CFU) under 50% N was 1.2 to 1.4 times for SECS and 1.1 to 1.2 times for whole slurry than that under 0% N. Although the nutrients concentration increased from 50 to 100% N, ethanol concentration (Figure 2C and 2D) and cell viability (CFU) (Figure 3C and 3D) did not increase further. The reason for this result may be that high nutrients contained high salt concentration in high glucan loading fermentation broth, which may have inhibited cell viability and thereby reduce the SSF performance [31,32]. In addition, the high nutrients addition should also increase the costs of SSF. Therefore, results suggested that 50% N addition should meet the requirements of SSF.

Initial pH is one of most important parameters for enzyme activity and cell viability in SSF [31,33]. Figure 2E and 2F shows that the highest initial ethanol yield was obtained at initial pH 4.8, which, at initial pH values of 4.0 and 5.5, was 1.41 times and 1.31 times that for SECS and 1.24 times and 1.08 times that for whole slurry, respectively. The highest final ethanol concentration was also obtained at initial pH 4.8, which was 28.6 g/L for SECS and 27.6 g/L for whole slurry. It is interesting to note that the highest cell viability was obtained at initial pH 5.5, followed by initial pH 4.8 and initial pH 4.0 (Figure 3E and 3F). Previous studies had reported that *S. cerevisiae* increased ethanol production at pH 5.0 and 5.5 as opposed to pH 4.0 and 4.5, and its optimum pH was from 5.0 to 5.2 [33]. Therefore, judging from the initial ethanol yield and final ethanol concentration, initial pH 4.8 was the optimum pH for SSF among the tested values.

Pre-hydrolysis time directly influenced the content and composition of unhydrolyzed solids, as well as the composition of hydrolysate in SSF, due to the different temperature between hydrolysis and fermentation [26,27,34]. The purpose of pre-hydrolysis of SECS in SSF was to hydrolyze the solid quickly at a high temperature so that a more homogenous and a higher glucose concentration hydrolysate could be formed for subsequent ethanol fermentation. In addition, pre-hydrolysis resulted in the low initial viscosity of fermentation broth due to the hydrolysis of the pretreated solid, so diffusion and mixing limitations could be minimized or altogether avoided during fermentation. The results show that glucose concentrations after 24, 36, and 48 hours pre-hydrolysis were 45.1, 49.0, and 51.3 g/L for SECS and 43.8, 47.1, and 49.6 g/L for whole slurry, respectively (Figure 2G and 2H). It was clearly observed that the order effects of pre-hydrolysis time on initial ethanol yield and final ethanol concentration were 48 hours, followed by 36 hours, followed by 24 hours. The yeast cell viabilities were also obviously enhanced with the increase of pre-hydrolysis time due to the fact that the increased initial glucose concentration provided more nutrients (Figure 3G and 3H). However, it should be noted that the SSF performance for 36 hours was almost similar to that for 48 hours, and the increase of pre-hydrolysis time may obviously increase the sugar’s inhibitory effect and reduce the utilization efficiency of equipments. Therefore, the optimum pre-hydrolysis time was determined as 36 hours in SSF.

DPs formed by pretreatment (such as carboxylic acids, phenols, and furans) were considered as the potential inhibitors in high glucan loading fermentation [18,20,35]. DPs’ concentrations in steam-exploded liquor (SEL) were 2.3 g/L formic acid, 2.7 g/L acetic acid, 0.7 g/L HMF, and 1.0 g/L furfural (Table 1). In this study, a novel *S. cerevisiae* which can tolerate 5.3 g/L acetic acid, 1.3 g/L furfural, and 0.5 g/L phenol [36], was used to reduce the DPs’ inhibitory effect. The results implied that initial ethanol yields of whole slurry (Figure 2B, 2D, 2F and 2H) were 5 to 15% lower than that of SECS (Figure 2A, 2C, 2E and 2G), but it was clearly observed that ethanol concentration of whole slurry was approximate to that of SECS after 48 hours fermentation under the same fermentation conditions. Figure 3 show that approximately the highest cell viability was obtained at 12 hours for SECS (Figure 3A, 3C, 3E and 3G) and at 24 hours for whole slurry (Figure 3B, 3D, 3F and 3H) in SSF. However, cell viability (CFU) for whole slurry was approximate to that for SECS, with fermentation progression. Therefore, the results suggest that the DPs’ concentration appears to be too low to impair the fermentation performance, indicating that thermal- and ethanol-tolerant *S. cerevisiae* had good fermentability of either washed SECS or whole slurry.

The products’ (sugar) feedback inhibitory effect was also a major problem for the traditional SHF process. In the SSF process, the increase of viability (CFU) was observed in the first 12 hours fermentation for SECS and 24 hours fermentation for whole slurry (Figure 3), due to the high initial glucose concentration (Figure 2). Glucose was still being released after 24 hours fermentation, but the cell viabilities began to decrease. The possible reason for this result may be that the glucose release rate was too slow to satisfy the demand of yeast cell growth. Glucose concentration was less than 2.0 g/L for SECS and 3.0 g/L for whole slurry after 6 hours fermentation, but ethanol concentration still increased with fermentation progression, which indicated that the sugar’s inhibitory effect should be removed in SSF at high glucan loading.

### Effects of temperature on the simultaneous saccharification and fermentation performance

Traditional SSF (conducted at 30°C) shortened the overall process time by combining hydrolysis and ethanol fermentation, and hence had a higher productivity than SHF. However, the low processing temperature limited the sugar conversion and hence limited the further improvement of ethanol productivity in SSF [17,27]. In general, the increase of temperature increased sugar conversion and the increase of ethanol concentration decreased sugar conversion in the traditional SSF process. The ideal temperature is approximately 30°C for most *S. cerevisiae* strains and 50°C for cellulases. A higher temperature should stress *S. cerevisiae* and make them more susceptible to other stresses such as ethanol, especially at ethanol concentration beyond 30 g/L [33,37]. Therefore, thermal- and ethanol-tolerant yeast strains for improving ethanol productivity are required to fully exploit the merits of the SSF process.

**Figure 4:**
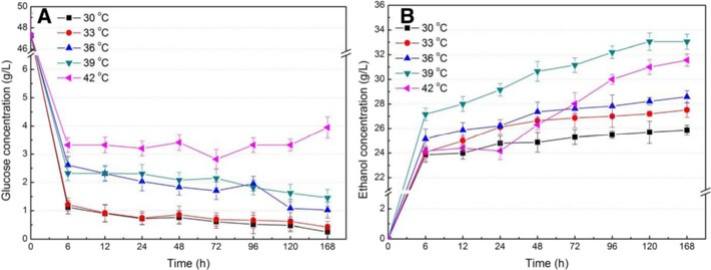
**Simultaneous saccharification and fermentation performances of steam-exploded corn stover at different temperatures.** Glucose **(A)** and ethanol **(B)** concentration were determined in SSF at different temperatures. The experiments were conducted at inoculation OD 4.0, pH 4.8, 50% nutrients, 36 hours pre-hydrolysis time, and 6% glucan loading. OD, optical density; SSF, simultaneous saccharification and fermentation.

Thermal- and ethanol-tolerant *S. cerevisiae*, which can tolerate up to 42°C and 6% (w/v) ethanol, was used in SSF of SECS at inoculation OD 4.0, initial pH 4.8, 50% nutrients, 36 hours pre-hydrolysis time, and 6% glucan loading (Figure 4). The results show that glucose concentration at 6 hours fermentation was less than 1.0 g/L at 30°C and 33°C, and approximate 2.0 g/L at 36°C and 39°C, while it was about 3.0 g/L at 42°C (Figure 4A). It indicated that glucose concentration was maintained at a low level in SSF, which obviously reduced the products’ feedback inhibitory effect. It is interesting to note that ethanol concentration increased by 12.6% for 30°C, 15.8% for 33°C, 15.0% for 36°C, 22.5% for 39°C, and 33.7% for 42°C, with fermentation time increasing from 6 to 168 hours (Figure 4B). Compared with that for 30°C (traditional SSF), initial ethanol yield increased by 1.0% for 33°C, 5.9% for 36°C, 14.3% for 39°C, and 1.7% for 42°C at 6 hours, and final ethanol concentration increased by 6.4%, 10.6%, 27.8%, and 22.7%, respectively. It should be noted that ethanol concentration hardly changed at 42°C with fermentation time varying from 6 to 24 hours, and then rapidly increased with an increase of fermentation time. The reason for this result may be that *S. cerevisiae* adapts to the high temperature environment at the beginning of fermentation. The maximal initial ethanol yield and final ethanol concentration were obtained at 39°C. These results indicated that the SSF performance of SECS was improved with the temperature increasing from 30°C to 39°C using thermal- and ethanol-tolerant *S. cerevisiae*. Therefore, the optimal 39°C was a chosen as a compromise for increasing the activity of cellulase and allowing *S. cerevisiae* to still ferment sugars in SSF.

By-products of fermentation were the major factor for evaluating SSF performance. Effects of fermentation conditions on by-products (glycerol and acetic acid) in SSF of washed SECS and whole slurry were carried out, and the results are given in Table 3. The glycerol concentration varied from 0.84 to 1.64 g/L for washed SECS, while the acetic acid concentration varied from 1.94 to 3.21 g/L under different SSF conditions. It is interesting to note that the glycerol concentration varied from 1.23 to 1.87 g/L for whole slurry, and the acetic acid concentration varied from 3.01 to 4.52 g/L under different SSF conditions, which increased by 5.6 to 19.8% and 20.6 to 65.1% compared with that for SECS, respectively. The possible reason for these results should be that whole slurry contained the DPs formed in pretreatment, which enhanced the secondary metabolism of *S. cerevisiae*. Furthermore, the acetic acid generated from pretreatment increased the final acetic acid concentration in SSF of whole slurry. However, the ethanol concentration for whole slurry decreased by only 2 to 4%, compared with that for washed SECS. These results indicated that the thermal- and ethanol-tolerant *S. cerevisiae* had the good fermentability of washed SECS and whole slurry in SSF at high temperature.

**Table 3:** **Effects of inoculation OD, nutrients, initial pH, pre-hydrolysis time, and temperature on glycerol, acetic acid, and ethanol production in the SSF process of washed SECS and whole slurry by** ***Saccharomyces cerevisiae***

	**Washed SECS**			**Whole slurry (SECS + SEL)**		
**Experimental parameters**	**Glycerol (g/L)**	**Acetic acid (g/L)**	**Ethanol (g/L)**	**Glycerol (g/L)**	**Acetic acid (g/L)**	**Ethanol (g/L)**
Initial OD_600_						
1.0	1.21 (0.04)	2.34 (0.08)	27.3 (0.7)	1.45 (0.06)	3.01 (0.09)	26.2 (0.7)
4.0	1.25 (0.04)	2.72 (0.06)	28.6 (0.8)	1.37 (0.05)	3.56 (0.08)	27.7 (0.9)
8.0	1.51 (0.06)	2.83 (0.04)	26.7 (0.8)	1.74 (0.04)	4.28 (0.04)	24.6 (0.8)
Nutrients						
0%	1.17 (0.05)	1.96 (0.05)	26.4 (0.6)	1.29 (0.04)	3.05 (0.05)	27.1 (0.6)
50%	1.25 (0.04)	2.72 (0.06)	28.6 (0.8)	1.37 (0.05)	3.56 (0.08)	27.7 (0.9)
100%	1.62 (0.03)	2.48 (0.09)	25.7 (1.1)	1.91 (0.04)	4.34 (0.07)	25.1 (1.0)
Initial pH						
4.0	0.84 (0.07)	1.94 (0.05)	25.3 (0.3)	1.32 (0.07)	3.03 (0.06)	26.2 (0.6)
4.8	1.25 (0.04)	2.72 (0.06)	28.6 (0.8)	1.37 (0.05)	3.56 (0.08)	27.7 (0.9)
5.5	1.61 (0.03)	2.86 (0.08)	25.4 (0.7)	1.87 (0.08)	4.01 (0.09)	24.8 (1.2)
Effects of pre-hydrolysis time						
24 hours	1.08 (0.06)	2.77 (0.09)	25.5 (0.7)	1.23 (0.07)	3.64 (0.06)	24.9 (0.7)
36 hours	1.25 (0.04)	2.72 (0.06)	28.6 (0.8)	1.37 (0.05)	3.56 (0.08)	27.7 (0.9)
48 hours	1.56 (0.04)	2.32 (0.05)	27.6 (0.5)	1.71 (0.04)	3.34 (0.05)	25.9 (0.6)
Effects of temperature						
30°C	1.12 (0.06)	3.01 (0.08)	25.8 (0.8)	1.23 (0.03)	4.28 (0.09)	24.9 (0.4)
33°C	1.31 (0.04)	3.21 (0.06)	27.5 (1.2)	1.46 (0.06)	4.52 (0.06)	26.5 (0.5)
36°C	1.25 (0.04)	2.72 (0.03)	28.6 (0.8)	1.37 (0.05)	3.56 (0.08)	27.7 (0.9)
39°C	1.64 (0.07)	2.52 (0.06)	33.1 (0.6)	1.73 (0.07)	3.04 (0.07)	32.1 (0.8)
42°C	1.55 (0.03)	2.24 (0.07)	31.5 (1.3)	1.69 (0.09)	3.01 (0.05)	30.2 (1.0)

Standard deviations are shown in parentheses. OD, optical density; SECS, steam-exploded corn stover; SEL, steam-exploded liquor; SSF, simultaneous saccharification and fermentation.

### Effects of glucan loading on the simultaneous saccharification and fermentation performance

**Figure 5:**
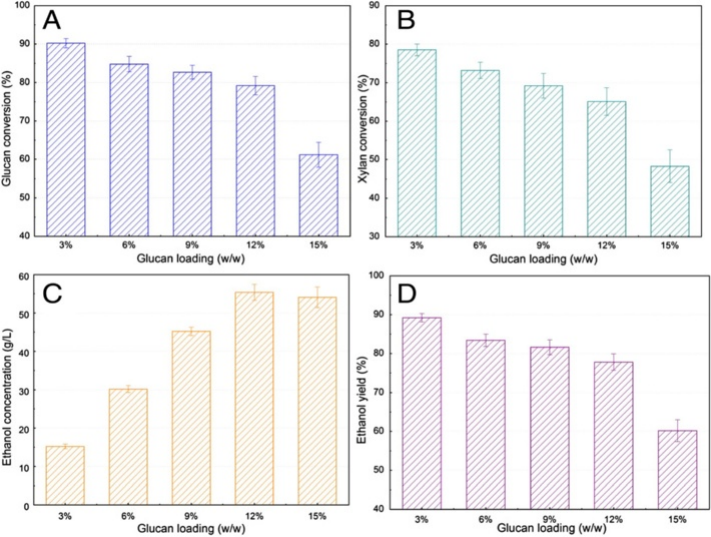
**Simultaneous saccharification and fermentation performances of steam-exploded corn stover at different glucan loadings.** Glucan conversion **(A)**, xylan conversion **(B)**, ethanol concentration **(C)**, and ethanol yield **(D)** were determined in SSF. The experiments were conducted at inoculation OD 4.0, pH 4.8, 39°C, 50% nutrients, and 36 hours pre-hydrolysis time. OD, optical density; SSF, simultaneous saccharification and fermentation.

An important factor in the process economics and energy balance is the concentration of glucan loadings in the stream entering the hydrolysis and fermentation step [9,19,24]. By increasing glucan loading, the resulting sugar concentration and, consequently, final ethanol concentration will be higher. This has a significant effect on production cost due to the reduced size of equipment (tanks and distillation column, and so forth) and the reduced energy utilization for distillation [18,20]. Effects of glucan loading (3 to 15% glucan loading, corresponding to 5.0 to 25.4% solid loading) on the SSF performance of SECS at optimized conditions were carried out. The results show that 3% glucan loading showed the highest glucan conversion (Figure 5A), xylan conversion (Figure 5B), and ethanol yield (Figure 5D), but it also obtained the lowest ethanol concentration (Figure 5C). With glucan loading increasing from 6 to 12%, glucan conversion, xylan conversion, and ethanol yield reduced less than 5.6%, 8.0%, and 5.2%, respectively, compared with that at 3% glucan loading. Ethanol concentration increased from 30.2 to 55.4 g/L when glucan loading increased from 6 to 12%, and the relative ethanol concentration increased by 83.4%.

Previous research has stated that the most economical ethanol distillation process should be achieved when the ethanol concentration was more than 4% (w/w) [18,19,25]. Ethanol concentration reached 45.2 and 55.4 g/L at 9% and 12% glucan loading (Figure 5C), respectively, with high glucan conversion and ethanol yield, which implied that SSF of SECS at high glucan loading and high temperature should meet the requirements of ethanol industrial production. It should be noticed that although ethanol concentration was approximate to that at 12% glucan loading, glucan conversion, xylan conversion, and ethanol yield rapidly decreased at 15% glucan loading (25.4% solid loading) (Figure 5). The possible reason for this phenomenon may be that the increased solid content increased the viscosity of the mixture and hence reduced the mixing efficiency, leading to poor mass and heat transfer in SSF [18,19,38]. The results suggested that the approximate boundary of mass transfer limitations for the SSF of SECS was about between 12% and 15% glucan loading, corresponding to 20.3% and 25.4% solid loading. Therefore, 9% and 12% glucan loading should be the suitable conditions for converting SECS to ethanol in SSF.

### Effects of surfactants on performance of simultaneous saccharification and fermentation

**Figure 6:**
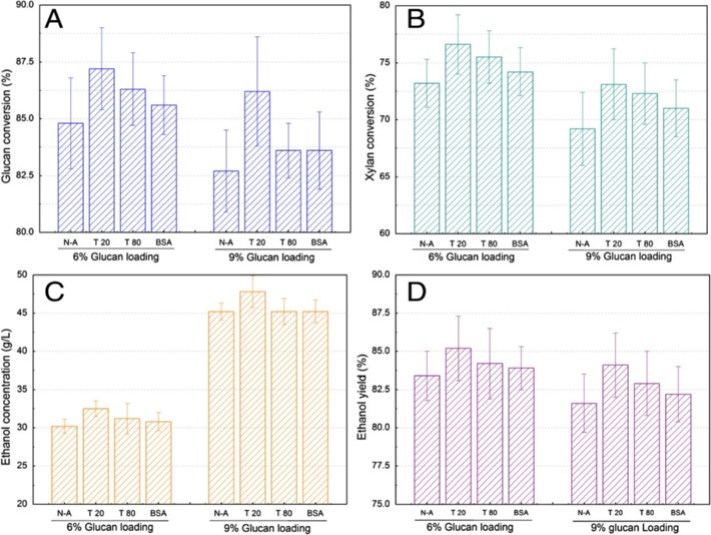
**Effects of adding 2.0% (w/w) Tween 20, Tween 80, and BSA on the SSF performance of SECS.** Glucan conversion **(A)**, xylan conversion **(B)**, ethanol concentration **(C)**, and ethanol yield **(D)** were determined in SSF. The experiments were conducted at inoculation OD 4.0, pH 4.8, 39°C, 50% nutrients, and 36 hours pre-hydrolysis time. N-A is Non-addition; T 20 and T 80 is Tween 20 and Tween 80, respectively. BSA, bovine serum albumin; OD, optical density; SSF, simultaneous saccharification and fermentation.

Surfactants have been proven to be effective in enhancing enzymatic hydrolysis of cellulose [39-41], but the effects of surfactants on the SSF of SECS to ethanol at high glucan loading is not well understood. We explored the effects of the surfactants Tween 20, Tween 80, and BSA as an additive on the SSF performance at 6% and 9% glucan loading, corresponding to 10.2% and 15.3% solid loading (Figure 6).

It was clearly observed that the surfactants Tween 20, Tween 80, and BSA obviously increased glucan and xylan conversion, and hence increased ethanol concentration and ethanol yield, compared with no addition of surfactants at either 6 or 9% glucan loading (Figure 6). One possible reason for this phenomenon may be that surfactants prevented the unproductive binding of the cellulases to the fermentation residues. This allowed more enzyme to be available for the conversion of cellulose, resulting in a higher sugar conversion and hence a higher ethanol yield [39,40]. Another possible reason may be that surfactants increased enzyme stability by reducing the thermal denaturation effect and improving the properties of solvents, and hence the efficiency of enzyme-substrate interaction [39,40]. The inhibitory effect of unhydrolyzed solids was the major cause for the poor SSF performance, due to the binding of yeast cells to the solid residues [27]. Surfactants may also improve the cell viability by reducing the unproductive binding of cells to fermentation residues.

It is interesting to note that the highest glucan and xylan conversion was obtained with 2% Tween 20 addition, which increased by 3.8% and 4.6% at 6% glucan loading, and 4.3% and 5.6% at 9% glucan loading, respectively, compared to that without surfactants addition (Figure 6A and 6B). Furthermore, the highest glucose concentration and ethanol yield was also obtained with 2% Tween 20 addition, and they increased by 7.6% and 2.2% at 6% glucan loading and 5.5% and 3.1% at 9% glucan loading, respectively, compared to that without surfactants addition (Figure 6C and 6D). The results indicated that the addition of 2% Tween 20 obviously improved the SSF performance of SECS at 6% and 9% glucan loading under 39°C. A previous study also confirmed that ethanol yield increased by 8% in SSF of steam-pretreated softwood with the addition of Tween-20, and the enzyme activity increased in the liquid fraction at the end of SSF [41]. It should be noted that the industrial process of ethanol production should consider the relations of the cost and environmental effect of surfactants and improved SSF performance. Previous studies have indicated that the surfactants addition in SSF increased the capital cost of biomass conversion process, but surfactants addition reduced the enzyme loading and increased the fermentation performance [39-41]. Meanwhile, surfactants addition decreased the enzyme adsorption onto the solid residue, which should make enzyme recovery recycle from the SSF system possible [39-41]. These studies also suggested that the further work should be conducted to balance the capital cost of surfactant and the SSF performance.

### Mass balance comparison between separate hydrolysis and fermentation and simultaneous saccharification and fermentation

**Figure 7:**
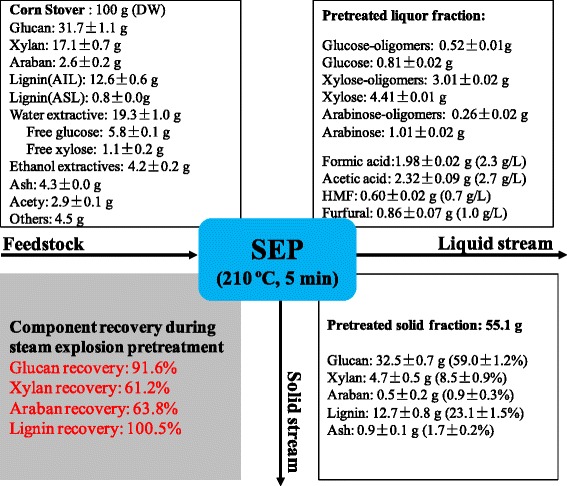
**Mass balance analysis for steam explosion pretreatment (SEP) of corn stover biomass at 210°C for five minutes.** SEP, steam explosion pretreatment; DW, dry weight.

**Figure 8:**
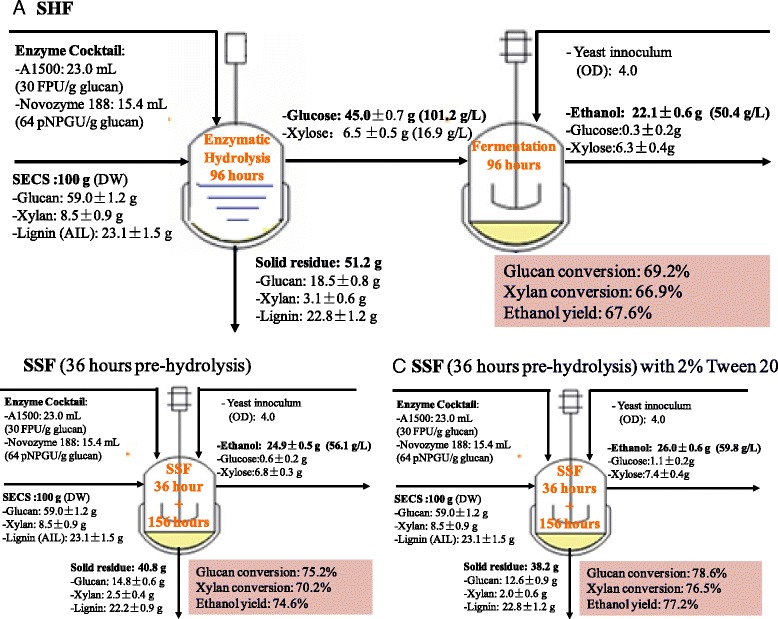
**Mass balance analysis for separate hydrolysis and fermentation (SHF) (A), simultaneous saccharification and fermentation (SSF) (B), and SSF with 2% Tween 20 (C).** For SHF, enzymatic hydrolysis was carried out at pH 4.8, 50°C, 200 rpm for 96 hours and fermentation of hydrolyzate was conducted at inoculation OD 4.0, 30°C, 50% nutrients, 200 rpm for 96 hours. For SSF, experiments were performed at 39°C, inoculation OD 4.0, pH 4.8, 50% nutrients, and 200 rpm for 192 hours. Data were collected from 12% (w/w) glucan loading experiments of SECS conversion. AIL, acid insoluble lignin; DW, dry weight; FPU, filter paper unit; OD, optical density; pNPGU, p-nitrophenol-*β*-D- glucopyranoside units; SECS, steam-exploded corn stover; SHF, separate hydrolysis and fermentation; SSF, simultaneous saccharification and fermentation.

Mass balance is essential to evaluating the biomass conversion process for LCE production [42-45]. For a systematical evaluation of CS conversion process, mass balance studies were performed on the processes of SEP, SHF, SSF, and SSF with 2% Tween 20 addition at the optimal conditions (Figures 7 and 8). As for SEP, Figure 7 shows that glucan recovery reached 91.6%, which is higher than that for different acid pretreatment at 190°C and 90 mM [42]. This indicated that SEP hardly degraded cellulose, which is confirmed by previous studies [4,12]. It should be noted that xylan and araban recovery was 61.2% and 63.8%, respectively, which implied that the hemicelluloses were partly degraded in SEP. The glucan content of SECS solid reached 59.0% and the acetyl of UCS was removed, which should facilitate the conversion process of CS.

Traditional SHF resulted in low sugar conversion in enzymatic hydrolysis and low ethanol yield in fermentation, likely due to the fact that the enzyme activity was inhibited by products’ feedback inhibitory effect. After 96 hours hydrolysis and 96 hours fermentation, a glucose concentration of 101.2 g/L and an ethanol concentration of 50.4 g/L were obtained at 12% glucan loading in SHF. This corresponded to a glucan conversion of 69.2% and an ethanol yield of 66.9% based on the maximum theoretical yield of glucose presented in SECS biomass. The low processing temperature (30°C) of traditional SSF limited the hydrolysis rate and hence limited the further improvement of ethanol productivity. Compared with traditional SHF and SSF, 36 hour pre-hydrolysis SSF at 39°C obviously increased glucan conversion from 69.2 to 75.2%, and hence increased ethanol yield from 66.9 to 70.2%. The relative glucan conversion and ethanol yield increased by 8.7% and 5.0%, respectively (Figures 8A and 8B). To remove the solid’s inhibitory effect on enzymatic hydrolysis and fermentation, 2% Tween 20 was added in SSF and mass balance was also conducted around the whole process (Figure 8C). Glucan conversion and ethanol yield reached 78.6% and 77.2%, respectively, and the ethanol concentration reached 59.8 g/L in 36 hour pre-hydrolysis SSF with 2% Tween 20 addition at 39°C. The relative glucan conversion and ethanol yield increased by 4.5% and 3.5%, respectively, compared with 36 hour pre-hydrolysis SSF at 39°C. These results indicated that high glucan loading and high temperature obviously improved the SSF performance. Therefore, the SSF of SECS at high glucan loading and high temperature using a novel thermal- and ethanol-tolerant *S. cerevisiae* should be an effective conversion process for ethanol production.

## Conclusions

The present results suggest that glucan conversion of SECS is maintained at approximately 71 to 79%, at an enzyme loading of 30 FPU/g glucan, with glucan loading varying from 3 to 12% in enzymatic hydrolysis. The optimal parameters of SSF include inoculation OD 4.0, initial pH 4.8, 50% nutrients, 36 hours pre-hydrolysis time, 39°C, and 12% glucan loading (20% solid loading). Under the optimal conditions, glucan conversion, ethanol yield, and final ethanol concentration of SSF reached 78.6%, 77.2%, and 59.8 g/L, respectively, with 2% Tween 20 addition. The inhibitory effects of DPs, products, and solids were not obvious in SSF at 6 to 12% glucan loading. Therefore, compared with traditional SHF and SSF, high glucan loading and high temperature obviously improved the SSF performance of SECS using a novel thermal- and ethanol-tolerant strain of *S. cerevisiae* in this study.

## Materials and methods

### Biomass source

CS biomass used for this study was collected from the suburb of Tianjin, China. CS was air-dried to the moisture content of 5 to 10%. For the composition analysis, CS was milled by knife mill (YS-08, BYZME, Beijing, China), and passed through a 20-mesh screen. The composition analysis was conducted using the Laboratory Analysis Protocol (LAP) of the National Renewable Energy Laboratory, Golden, Colorado, United States. Compositions of UCS are given in Table 1.

### Steam explosion pretreatment

SEP was conducted by a pretreatment unit consisting of a 15-L steam explosion reactor (Tianjin hanyang metal equipment Co., Ltd, Tianjin, China), a 150-L reception chamber (Tianjin hanyang metal equipment Co., Ltd, Tianjin, China), and a steam generator (Jinan Sanheng equipment Co., Ltd, Shandong, China), and followed our previous procedure [12,46]. SEP conditions included: temperature of 210°C, residence time of five minutes, and 30% initial moisture content. After pretreatment, SECS was separated from the liquid fraction by vacuum filtration using a Buchner funnel (Tianjin shunlongda technology Co., Ltd, Tianjin, China) and then washed with deionized water at solid-to-water ratio 1:15. Compositions of SECS and SEL were analyzed, and the results are shown in Table 1.

### Enzymatic hydrolysis

Accellerase 1500 was a generous gift from Genencor (Jiangsu, China). Novozyme 188 was purchased from Sigma-Aldrich (St Louis, Missouri, United States). The filter paper unit of Accellerase 1500 is 77 FPU/mL, while xylanase and *β*-xylosidase is 72 IU/mL and 23 IU/mL, respectively. The *β*-glucosidase activity of Novozyme 188 is 250 p-nitrophenol-*β*-D- glucopyranoside units (pNPGU)/mL. SECS was hydrolyzed at different glucan loadings in a 0.05 M citrate buffer solution (pH 4.8) with an Accellerase 1500 loading of 15 FPU, 30 FPU, or 60 FPU/g glucan and a *β*-glucosidase loading of 64 pNPGU/g glucan. The experiments were conducted at 50°C and 200 rpm for 168 hours. Hydrolyzate was collected by centrifuging at 10,000 rpm for 10 minutes. The residues were washed with a volume of water equal to 15 times the dry weight of initial SECS. Composition in hydrolyzate and washed liquid were analyzed using HPLC. All these experiments were conducted with two replicates. Glucan conversion was calculated based on that glucan dissolved into the liquor divided by glucan content in SECS.

### Microorganism and seed culture preparation

*S. cerevisiae*, angel thermal- and ethanol-tolerant alcohol active dry yeast (product number: 80000012, Angel Yeast Co. Ltd., Hubei, China), was used in this study. The acclimatization of this yeast strain was conducted by multiple rounds of the adaptive culture in inhibitors medium containing acetic acid, furfural, and phenol (Sinopharm Chemical Reagent Co.,Ltd, Shanghai, China). *S. cerevisiae* can tolerate 5.3 g/L acetic acid, 1.3 g/L furfural, and 0.5 g/L phenol [36]. For seed preparation, *S. cerevisiae* was cultivated in yeast extract peptone dextrose (YPD) medium (20 g/L glucose, 10 g/L yeast extract, and 20 g/L peptone) at 30°C and 150 rpm for 12 hours. The yeast cells were then inoculated to the secondary seed medium (20 g/L glucose, 10 g/L yeast extract, and 20 g/L peptone) and cultivated at 30°C and 150 rpm for 12 hours. Cell density was measured at 600 nm (1-cm light path) using a UV-vis spectrometer (Beckman Coulter Inc., California, United States). OD was corrected between 0.1 and 0.7 with the dilution factors as necessary. The initial OD for secondary seed culture was 0.05.

### Separate hydrolysis and fermentation

For SHF, washed SECS were pre-hydrolyzed with en enzyme loading of 30 FPU/g glucan at pH 4.8, 50°C, and 200 rpm for 96 hours. After pre-hydrolysis, the hydrolyzate was separated from the hydrolysis residues. Hydrolyzate was then transferred into 250 mL Erlenmeyer flasks capped with rubber stoppers with a working volume of 100 mL. Yeast cell pellets used for inoculation were obtained by centrifuging the seed culture at 5,000 rpm for 10 minutes. Fermentations of hydrolyzate were carried out at pH 4.8, 30°C, and 200 rpm for 96 hours. Fermentation samples were taken at different time-points and centrifuged at 10,000 rpm for 10 minutes. Supernatants were filtered through a 0.22-μm Whatman syringe filter (Shanghai Wanzi shiye Co., Ltd, Shanghai, China) and analyzed by HPLC. All experiments were conducted with two replicates.

### Simultaneous saccharification and fermentation

SSF experiments were conducted in 250-mL Erlenmeyer flasks with 100 mL of total mixture for 192 hours at 200 rpm. Experimental parameters for SSF of washed SECS and whole slurry are shown in Table 2. The enzyme loading used in SSF is 30 FPU/g glucan. In SSF, samples were taken at different time-points and centrifuged at 10,000 rpm for 10 minutes. Supernatants were filtered through a 0.22-μm Whatman syringe filter. Compositions of liquid fraction and fermentation residues were determined by HPLC. In an attempt to investigate the effects of surfactants on the SSF process, 2% (w/w) of Tween 20, Tween 80, and BSA (Tianjin Xiensi biochemistry technology Co., Ltd, Tianjin, China) were added at the beginning of SSF. All these experiments were conducted with two replicates. Ethanol yield in SHF and SSF was calculated based on the maximal theoretical ethanol yield from consumed glucose only, which is 0.51 g ethanol/g glucose. Initial ethanol yield was calculated based on ethanol content divided by the maximal theoretical ethanol content from glucose only, at the first 6 hours fermentation. Final ethanol yield and concentration were calculated at 168 hours fermentation. 100% N stands for 100% nutrients, which was defined as 10 g/L yeast extract and 20 g/L peptone; 50% N was defined as 5 g/L yeast extract and 10 g/L peptone; and 0% N was defined as 0 g/L yeast extract and 0 g/L peptone (Table 2).

### Measurement of viable cell density

Because it is impossible to measure the OD in SSF of washed SECS and whole slurry, CFU was measured to determine the viable cell density. Fermentation slurry was sampled and diluted using sterile water. 100 μL of each diluted sample was taken and plated on an YPD agar medium (20 g/L glucose, 10 g/L yeast extract, and 20 g/L peptone). The dilution rate for each sample was varied to guarantee that the number of colonies on a single plate was between 20 and 200. The plates were then cultured at 30°C for 48 hours. Single colonies formed on the plates were counted and viable cell density was calculated accordingly.

### Analysis methods and mass balance

Sugars, DPs, glycerol, acetic acid, and ethanol were analyzed using HPLC (Waters, Milford, Massachusetts, United States) with a Biorad Aminex HPX-87H column (Biorad, Hercules, California, United States). Column temperature was maintained at 65°C. Mobile phase (5 mM H_2_SO_4_) flow rate was 0.6 mL/min. Mass balance was performed around the whole conversion process of SECS including SEP, enzymatic hydrolysis and fermentation for SHF, and fermentation for SSF. Error bars in the present Tables and Figures represent the standard deviation of the replicates. For all significance tests, Student’s t-test was used, requiring a probability *P* <0.05 in order to be significant.

## Abbreviations

BSA: Bovine serum albumin
CFU: Colony forming unit
CS: Corn stover
DPs: Degradation products
FPU: Filter paper unit
HMF: 5-hydroxymethyl furfural
LCC: Lignin-carbohydrate complex
LCE: Lignocellulosic ethanol
OD: Optical density
SECS: Steam-exploded corn stover
SEP: Steam explosion pretreatment
SHF: Separate hydrolysis and fermentation
SSF: Simultaneous saccharification and fermentation
UCS: Untreated corn stover
YPD: Yeast extract peptone dextrose
